# National Habit of Drinking

**Published:** 1892-02

**Authors:** 


### NATIONAL HABIT OF DRINKING.

This is a matter which concerns women nearly as much as it concerns men. One of the saddest facts is that a not inconsiderable number of women do fall victims to alcohol ; and there are one or two points in which the vice is more dangerous for the weaker sex. It is commonly said that if a man reaches middle age without getting drunk it is not likely that he will ever become a drunkard ; but it is not so with women. Again, natural and proper feelings of shame make a woman prefer to drink in secret ; and a secret vice is always harder to give up than an open one. But it is from another point of view altogether that the subject chiefly interests women. It is the woman's part to watch against the first advances of this deadly foe, to put forth all her influence and all her wit to repel it, to make him who is attacked feel that he is not alone in the struggle ; and, alas ! it is sometimes her part to suffer all the pain and shame that falls to the lot of a drunkard's wife. There are some who look to the action of the Legislature as the one thing that can put down our national vice.

There is, however, one point to which the attention of legislatures might be directed. Looking to the dangers incurred by the wife and children of an habitual drunkard, it might be desirable to declare inveterate drunkenness to be a crime—to be punished by lengthened periods of forcible restraint, without the low diet and monotonous toil of prison life.

The system would undoubtedly prevent many crimes being committed, and would thus lessen the expense of our gaols ; and it might be urged with much reason that a drunkard's wife and children have a right to be protected by preventive measures against being beaten and murdered, just as householders have a right to have the streets patrolled by police to prevent their houses being entered by burglars.—*Ex.*

